# Metal Oxide Thin Films Prepared by Magnetron Sputtering Technology for Volatile Organic Compound Detection in the Microwave Frequency Range

**DOI:** 10.3390/ma12060877

**Published:** 2019-03-15

**Authors:** Artur Rydosz, Andrzej Brudnik, Kamil Staszek

**Affiliations:** Department of Electronics, AGH University of Science and Technology, Al. Mickiewicza 30, 30-059 Krakow, Poland; brudnik@agh.edu.pl

**Keywords:** metal oxide thin films, gas sensors, volatile organic compound detection, microwave frequency

## Abstract

Metal oxide thin films such as copper oxide, titanium dioxide, and tin dioxide deposited by magnetron sputtering technology were verified as a gas-sensitive layer in microwave-based gas sensors operated at 2.4 GHz. The developed gas sensors were tested at room temperature (23 °C) and 50% relative humidity (RH) under exposure to 0–200 ppm of selected volatile organic compounds (acetone, ethanol, and methanol) that are of high interest in industry and biomedical applications. The highest responses to acetone were obtained for CuO-based gas sensors, to ethanol for SnO_2_-based gas sensors, while for methanol detection both dioxides, SnO_2_ and TiO_2_, exhibited good sensitivity.

## 1. Introduction

Magnetron sputtering technology is a well-known method for metal oxide (MOX) thin film deposition [1,2,3,4,5]. It is widely used in research and development laboratories as well as in the industry lines. There are various modes in magnetron sputtering technology including DC (direct current), MF (medium frequency), RF (radio frequency), and their combinations [6,7,8,9,10]. In 1935, Penning suggested the use of magnetron sputtering for thin film deposition [11], and since then, homemade and commercially available deposition systems have been developed for MOX deposition. The investigation of the thin film-based gas sensors was started over 50 years ago [12], however, there is a continuous need for developing novel materials with improved 3-S parameters (sensitivity, selectivity, stability) for gas-sensing applications. Table 1 summarizes the selected metal oxides (deposited by magnetron sputtering) used in gas sensors with specific parameters such as target gas and operating temperature, which is one of the critical features of metal oxide-based gas sensors. The most common metal oxides used as gas-sensitive layers are SnO_2_, TiO_2_, WO_3_, ZrO_3_, and ZnO. Every year thousands of reports are published with the gas-sensing characteristics of such materials. They are also commonly used in commercially available gas sensors, for example, in Figaro sensors. 

The major methods utilized for gas-sensing measurements are based on resistive and capacitive changes [36,37]. Since the resistance of metal oxide thin films is high, a gas-sensor’s substrates consist of electrodes with different geometries and materials, including interdigital capacitors (IDC) [38,39,40]. As can be noticed in Table 1, the operating temperature of MOX-based gas sensors is in the range of 25–1000 °C; hence silicon and alumina are commonly used gas-sensor substrate materials. To overcome the high operating temperatures, nanostructure forms of metal oxides have been recently proposed for gas sensors [41,42,43,44,45,46,47,48,49]. Another measurement technique that can be conducted at room temperature is a measurement in the microwave frequency range. In such a setup, the target gas molecules interact with a thin film of sensitive material, which is deposited in a microwave circuit. When exposed to the target gas, the sensor changes its scattering parameters, which in turn are measured with the aid of dedicated measurement apparatus (multiport measurement system or vector network analyzer). Then, the measured variance of the measured scattering parameters (i.e., resonant frequency shift or transmission coefficient’s phase difference) can be used to estimate target-gas concentration. Generally, organic materials are used as a gas-sensing layers in microwave-based gas sensors, for example, phthalocyanines [50,51,52,53]. Microwave-based gas sensors with metal oxide thin films were first introduced in 2007 by Jouhannaud et al. [54], where various oxides (such as: SnO_2_, SrTiO_2_, TiO_2_, ZnSO_4_, and ZrO_2_) were utilized as gas-sensitive layers and applied for water, ethanol, and toluene detection. The detection method was based on the reflection coefficient changes of the coaxial cable in the 300 kHz–3 GHz range. In 2016, Bailly et al. [55] showed a microwave-based gas sensor with a sensitive layer composed of commercial TiO_2_ nanoparticles for ammonia detection. The developed sensors exhibited a high response in the 100–500 ppm concentration range with good reversibility and stability [55].

In this paper, three different metal oxides, namely, copper oxide, titanium dioxide, and tin dioxide are proposed and verified as gas-sensitive layers for microwave-based gas sensors operating at room temperature and at 2.4 GHz with high sensitivity to selected volatile organic compounds: Acetone, ethanol, and methanol in the 0–200 ppm concentration range. 

## 2. Materials and Methods 

### 2.1. Sputtering Technology

Metal oxide thin films, as gas-sensitive layers, were deposited using a magnetron sputtering technology. Metallic targets (Kurt Lesker, Hastings, East Sussex UK) of copper (Cu), titanium (Ti), and tin (Sn) with 50 mm diameters and 99.995% purity were used in a reactive sputtering mode. The specific parameters during the deposition were different, since three different deposition systems were utilized. The films’ thicknesses were measured post-process using a TalyStep profilometer, Taylor Hobson, Leicester, UK. 

#### 2.1.1. Copper Oxide 

The copper oxide (CuO) thin films were deposited based on the previously confirmed results for gas-sensing applications [56]. Figure S1 shows a photo of the deposition system. Briefly, the base vacuum and deposition vacuum were 5 × 10^−6^ and 3 × 10^−2^ mbar, respectively. The target to substrate distance was approximately 45 mm and deposition temperature was 100 °C. The sputtering was conducted in a pure oxygen atmosphere, which is not common for magnetron sputtering technology, where Ar/O_2_ mixtures with different ratios are used. In this case a metallic target was first presputtered to remove any contamination in pure Ar for 10 min at 100 W, then pure O_2_ was introduced into the chamber and Ar was switched off. The films were obtained with the utilization of DC–MF sputtering mode. The CuO presputtering was continued for 30 min to stabilize the sputtering conditions. After the presputtering processes, the sputtering time was adjusted to obtain films with different thicknesses, the power, temperature, and oxygen flow were fixed at: 50 W, 100 °C, and 20 sccm, respectively.

#### 2.1.2. Titanium Dioxide 

Titanium dioxide thin films were deposited onto substrates using a homebuilt DC magnetron system (AGH University, Krakow, Poland) (Figure S2). The distance between the target and grounded substrate holder was kept at 50 mm. The deposition temperature was 200 °C, and it was made with external heating, using a stabilized resistive heater (AGH University, Krakow Poland) High-purity argon and oxygen were used as the sputtering and reactive gases, respectively. The base pressure of the system was better than 5 × 10^−6^ mbar, and the working pressure was kept at 1 × 10^−2^ mbar. The magnetron current was stabilized at I = 2 A, sputtering power varied from 0.9 to 1.0 kW. The discharge properties were controlled using optical emission spectroscopy (OES, AGH University, Krakow, Poland) [57], where the plasma light was collected with a quartz optical fiber through a window of the sputtering chamber (AGH University, Krakow, Poland). The optical emission Ti (500 nm) line intensity in the pure argon discharge was used as reference (I_0_) for monitoring the OES system. The oxygen flow was controlled to set the current titanium emission line intensity (I) to the desired value. The ratio I/I_0_ corresponds to the relative sputtering rate. The controlled oxygen flow was in the range of 1.5–0.9 sccm.

#### 2.1.3. Tin Dioxide 

The tin dioxide (SnO_2_) thin films were deposited in RF mode from a Sn metallic target. The base vacuum and deposition vacuum were 1 × 10^−5^ and 2 × 10^−2^ mbar, respectively with constant flow of an Ar/O_2_ mixture of 20% oxygen. The deposition temperature was set to 200 °C and deposition time was adjusted to deposit various thicknesses with a constant power of 50 W. The presputtering of metallic and oxide phases was 10 and 30 min, respectively. Figure S3 shows the deposition system for SnO_2_.

### 2.2. X-ray Diffraction 

The crystallographic structure of the metal oxide films was determined with X-ray diffraction by an X’Pert PRO MPD PANalytical system (Malvern Panalytical Ltd., Malvern, UK) with Cu Kα_1_ radiation. 

### 2.3. Scanning Electron Microscopy

The morphology of the metal oxide thin films was examined by scanning electron microscopy, FEI Versa 3D DualBeam (FEI, Hillsboro, OR, USA).

### 2.4. Gas-Sensing Measurements

**Figure 1:**
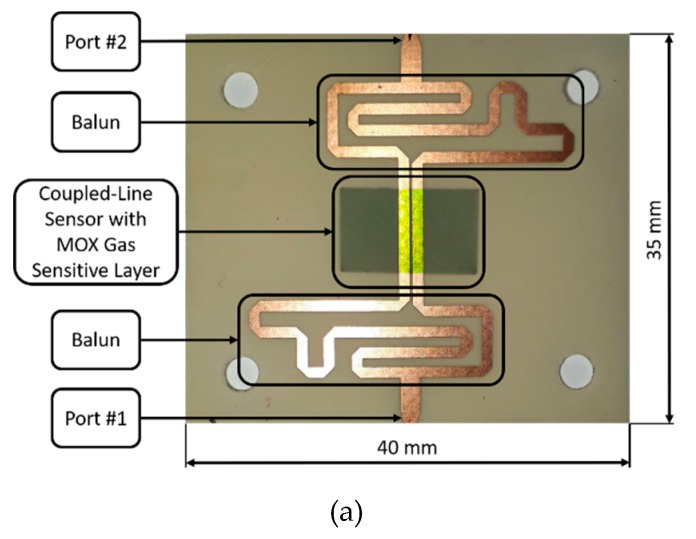

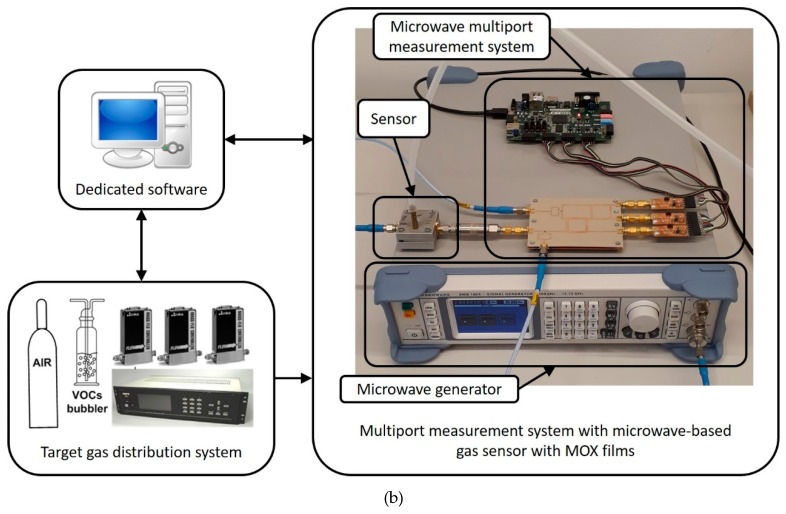
(**a**) A photograph of the utilized microwave gas sensor with metal oxide (MOX) film and (**b**) a schematic view of the entire gas-sensing measurement setup.

The gas-sensing behavior of the deposited metal oxide thin films was verified under exposure to selected volatile organic compounds in the 0–200 ppm range. Acetone, ethanol, and methanol were used due to their industrial [58] and biomedical applications [59,60]. The various concentration levels were obtained by utilization of mass flow controllers (MKS Instruments, Andover, MA, USA), Dreschel bottles (filled with pure solutions of VOCs), and controlled by mass spectrometer (HidenAnalytical, Warrington, United Kingdom), the gas-sampling system was previously described in [50]. The gas-sensing measurements were performed at room temperature and 50% relative humidity. The utilized microwave gas sensor () is illustrated in Figure 1a. It was realized as a single coupled-line section with proper feeding components, i.e., baluns ensuring an advantageous electromagnetic field distribution in the close proximity of the coupled section, which enhanced the sensor’s sensitivity [61]. The coupled-line section was covered with an MOX thin film, which when exposed to gas changed the relative permittivity, and therefore, the phase of the transmission coefficient between ports #1 and #2. The sensor was designed for the frequency 2.4 GHz using an Arlon 25N microwave substrate (Cirexx Internatioanl Inc, Santa Clara, CA, USA) and was enclosed in a metal housing with a channel allowing for gas flow and reaction with the MOX film. The mentioned sensor’s transmission coefficient was measured with the aid of the microwave multiport measurement system reported recently in [61]. It was composed of a signal source, power distribution network, the sensor, and three power meters. The signal delivered from the source was transmitted through the sensor and reference path; then both signals were correlated and provided to three power detectors, the readings of which were translated into the measured complex transmission coefficient (both magnitude and phase). The gas-sensor’s response was defined as a phase difference Δφ = φ_gas_ − φ_air_, where φ_gas_ and φ_air_ are transmission coefficient’s phases measured at the frequency of 2.4 GHz under exposure to target gas and air, respectively. A photograph of the entire measurement setup is shown in Figure 1b.

## 3. Results and Discussion

### 3.1. XRD Results

**Figure 2:**
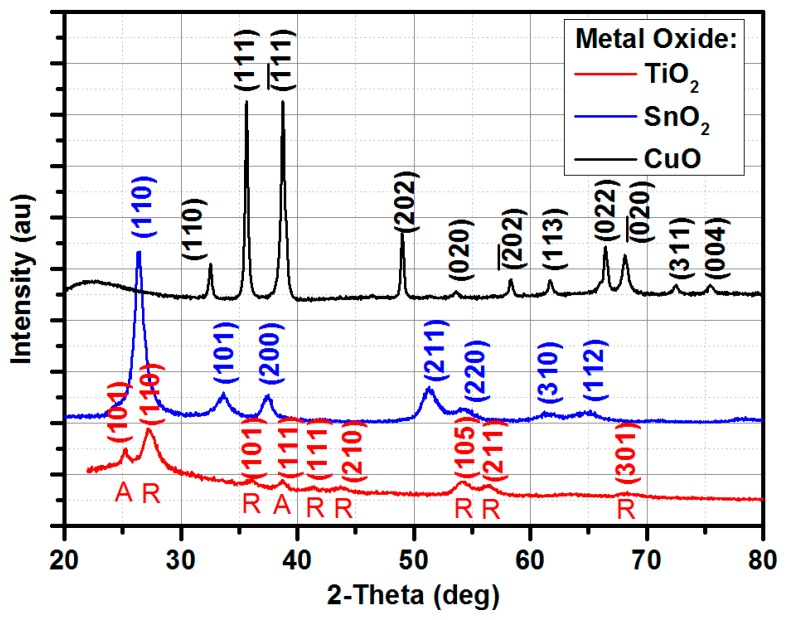
XRD diffractograms of the metal oxides: CuO, TiO_2_ (A–anatase, R–rutile), and SnO_2_ utilized as a gas-sensitive layer in microwave-based gas sensors.

Figure 2 shows the X-ray diffraction experiment results. The metal oxides were deposited on the microwave circuits, however, the XRD measurements were conducted on the reference samples deposited on a quartz substrate to avoid peaks from copper lines in the gas-sensor substrate. The CuO reflections were consistent with JCPDS data (48-1548) of the CuO with a monoclinic phase. It can be noticed that all peaks were related to CuO and no peaks of any impurities such as Cu/Cu_2_O were observed, indicating that a pure phase of CuO was deposited. In fact, the CuO thin films were deposited at a pure reactive mode (only oxygen was introduced into the deposition system). The SnO_2_ reflections were consistent with JCPDS data (41-1445) of the SnO_2_ with a tetragonal phase. The TiO_2_ diffractogram shows peaks from anatase and rutile phases, JPCDS data 21-1272 and JPCDS data 72-7374, respectively. 

### 3.2. SEM Results

**Figure 3:**
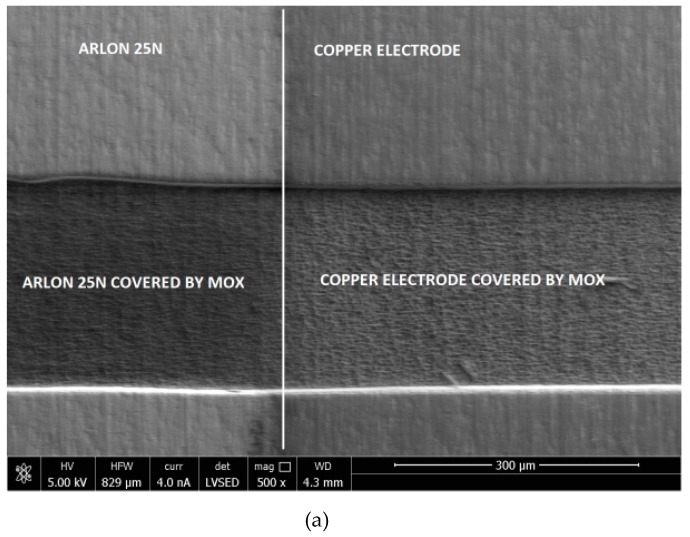

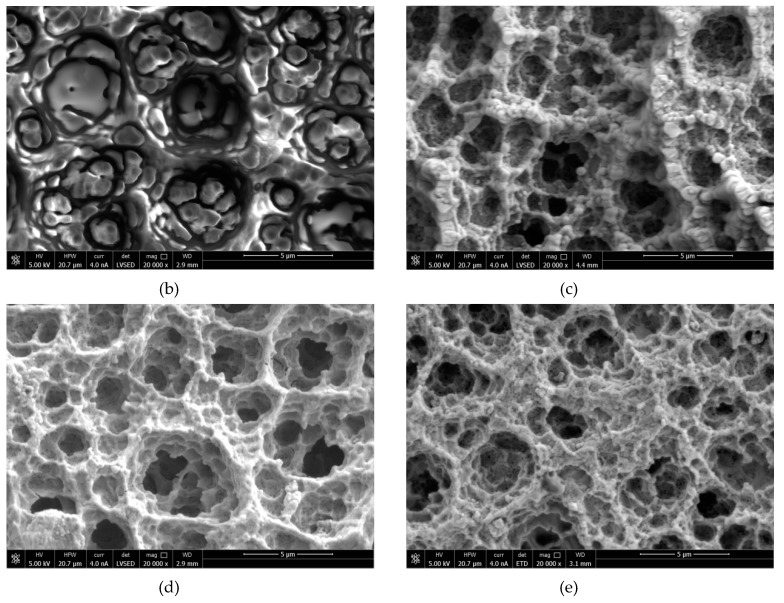
(**a**) SEM image: 500× magnitude of the microwave-based gas sensor with MOX gas-sensitive layer where pure substrate (Arlon 25N) and copper electrode with and without MOX gas-sensitive layer are shown; (**b**) SEM image: 20,000× magnitude of Arlon 25N substrate without MOX gas-sensitive layer; SEM image: 20,000× magnitude of substrate covered by TiO_2_ (**c**), SnO_2_ (**d**), and CuO; (**e**) gas-sensitive layers.

Figure 3a shows an SEM photo (500× magnitude) of the microwave-based gas sensor covered by metal oxide layer where pure substrate (both: Laminate and copper line) and substrate covered by metal oxide layer are presented. Figure 3b shows the 20,000× magnitude of the Arlon 25N structure without any gas-sensitive layer, and Figure 3c–e presents substrate covered by TiO_2_, SnO_2_, and CuO thin films, respectively. As can be noticed, the laminate substrate Arlon 25N is a highly porous structure, which makes it a very attractive material for gas-sensing applications, and when covered by selected metal oxides provides a high surface to volume ratio.

### 3.3. Gas-Sensing Results

#### 3.3.1. Thickness Selection

**Figure 4:**
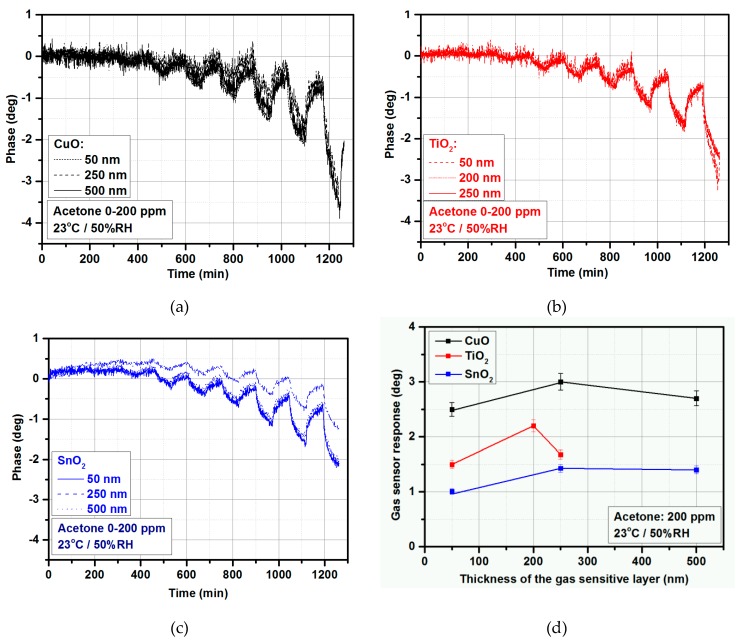
Gas-sensing characteristic under exposure to acetone in 0–200 ppm concentration range: (**a**) For CuO, (**b**) for TiO_2_, and (**c**) for SnO_2_; (**d**) gas-sensor response vs. thickness of the gas-sensitive layer at 200 ppm of acetone, 23 °C, and 50% relative humidity (RH).

**Figure 5:**
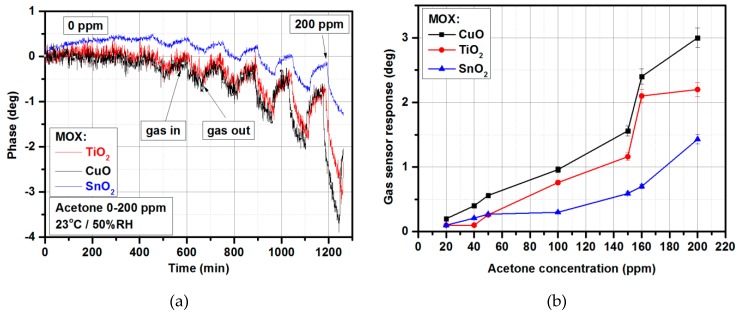
Acetone-sensing characteristics for microwave-based gas sensor with CuO, TiO_2_, and SnO_2_ thin films: (**a**) Phase changes at various acetone concentrations (23 °C/50% RH); (**b**) gas-sensor response vs. acetone concentrations.

**Figure 6:**
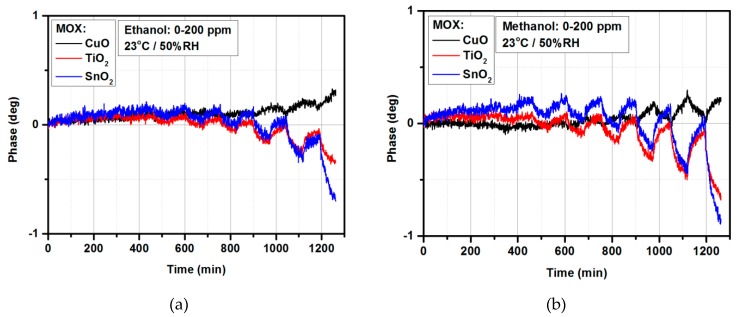
Alcohol-sensing characteristics for microwave-based gas sensor with CuO, TiO_2_, and SnO_2_ thin films: (**a**) Under exposure to ethanol; (**b**) under exposure to methanol.

**Figure 7:**
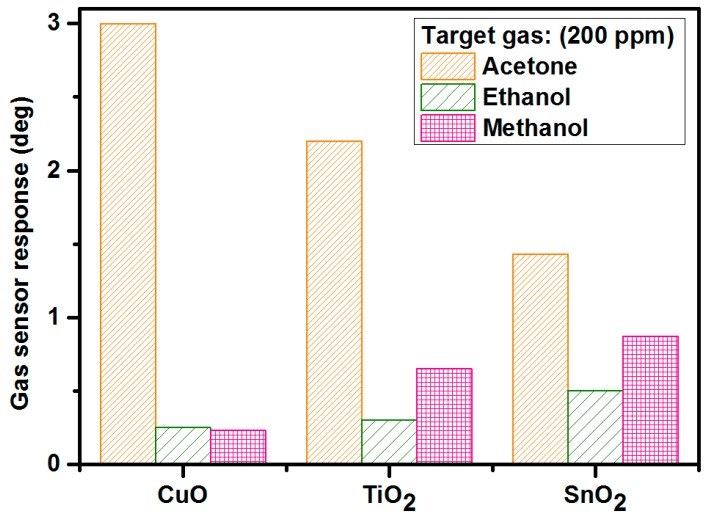
Gas-sensor response to acetone, ethanol, and methanol at room temperature, 50% relative humidity, and 200 ppm for CuO-based, TiO_2_-based, and SnO_2_-based microwave gas sensors.

The gas-sensing measurements were first conducted to select the optimum thickness of the deposited metal oxide thin films for gas sensors in the microwave frequency range. The presented transmission coefficient’s phase change of the sensor results from the fact that the target gas interacts with the sensing layer and changes its permittivity, which in turn affects the measured transmission coefficient. Figure 4 shows the gas-sensing results of CuO (Figure 4a), TiO_2_ (Figure 4b), and SnO_2_ (Figure 4c) under exposure to acetone at various concentrations in time. Gas in/gas out time slots are presented in Figure 5, Figure 6 and Figure 7. Figure 4d shows the comparison between the gas-sensor response and MOX thicknesses at a constant acetone concentration (200 ppm). As can be seen, the highest responses were obtained for 250 nm of CuO (~3 deg), 200 nm of TiO_2_ (~2.2 deg), and 250 nm of SnO_2_ (~1.43 deg). It has to be underlined that the obtained results were the starting points for further experiments and the optimal thicknesses could differ from the proposed ones. However, it is obvious that the optimal thickness was strictly dependent on the material; for copper oxide and titanium dioxide it was close to 250 and 200 nm, respectively; for tin dioxide, the gas-sensor responses for 250 and 500 nm varied only by ~0.03 deg, so the optimal thickness could be somewhere between 250 and 500 nm.

#### 3.3.2. Acetone Detection

Acetone is a colorless, mobile, flammable liquid readily soluble in water, ethanol, ether, etc., and itself serves as an important solvent. Acetone is an irritant, and its inhalation at higher concentrations may lead to hepatotoxic effects (causing liver damage) [62]. However, acetone can also be found in the exhaled human breath. The exhaled concentrations vary from 0.2 to 1.8 ppm for healthy people and from 1.25 to 2.5 ppm for people with diabetes [63]. Nowadays, the detection of acetone has become more and more attractive in the lower concentration range, since it is considered to be a biomarker of diabetes and exhaled acetone detection could replace blood glucose tests in the near future. Figure 5 shows the acetone-sensing characteristics for a microwave-based gas sensor with metal oxide gas-sensitive layers under exposure to acetone in the 0–200 ppm range (23 °C and 50% relative humidity (RH)). The time interval between gas in and gas out steps was constant and set to 90 min. As can be observed (Figure 5b) in the 0–200 ppm concentration range, the highest responses were obtained for CuO-based gas sensors. However, these sensors exhibited a phase drift (Figure 5a), and gas-sensor response (Figure 5b) calculated as a phase difference Δφ (Section 2.4) was affected by this drift. 

#### 3.3.3. Ethanol/Methanol Detection

Ethanol has bactericidal activity and is often used as a topical disinfectant. It is a clear and colorless liquid; widely used as a solvent and preservative in pharmaceutical preparations. It is also a primary ingredient of alcoholic beverages [64]. Methanol is the simplest alcohol and is a light, volatile, colorless, flammable, poisonous liquid. It is responsible for accidental, suicidal, and epidemic poisonings. The rapid and accurate diagnosis of toxic alcohol poisoning due to methanol (methyl alcohol) is paramount in preventing adverse outcomes. The quantitative measurement of specific serum levels of methanol using gas chromatography is expensive, time consuming, and generally only available at major tertiary-care facilities [64]. Rapid and portable methanol detectors are still expected by medical personnel. Figure 6 shows the alcohol-sensing characteristics for ethanol and methanol detection, Figure 6a,b, respectively. 

The CuO-based gas sensor exhibited a very low response to ethanol and methanol in the 0–200 ppm range (below 0.3 deg). TiO_2_ and SnO_2_ thin films were applied for ethanol and methanol detection; however, response to methanol was approximately twice higher than to ethanol in the 0–200 ppm range at room temperature and 50% relative humidity. Furthermore, the response to methanol was stable without the phase drift that was observed during the ethanol and acetone measurements. In order to avoid accidental results, the measurements were carried out three times starting from different gases. What is more, CuO is p-type and TiO_2_ and SnO_2_ are n-type semiconductor materials, however, there was no difference in the response in the microwave range. 

#### 3.3.4. Selectivity

Figure 7 presents the values of the gas-sensor response of the CuO-based (250 nm), TiO_2_-based (200 nm), and SnO_2_-based (250 nm) microwave gas sensors under exposure to various volatile organic compounds: Acetone, ethanol, and methanol. Target-gas concentration, measurement temperature, and relative humidity level were as follows: 200 ppm, 23 °C, and 50%. As can be noticed, the highest responses to acetone were obtained for CuO-based (~3), to ethanol for SnO_2_-based (~0.87), and to methanol for SnO_2_-based (~0.5) microwave gas sensors. Although the obtained phase differences were not large, they were at measurable levels, since the noise standard deviation was equal to ~0.04°. The copper oxide as a gas-sensitive layer exhibited a high sensitivity to acetone and practically no sensitivity to ethanol and methanol (below 0.3 deg of phase changes), which makes this metal oxide a very attractive gas-sensing material in microwave applications. The titanium dioxide and tin dioxide were used for methanol detection with the same response level.

## 4. Conclusions

Metal oxides are commonly used in gas-sensing applications as a gas-sensitive layer in resistive type sensors. In such a situation, higher operating temperature is generally required. In this paper, we have presented the investigation results of selected metal oxides, such as, copper oxide, titanium dioxide, and tin dioxide deposited by magnetron sputtering technology for volatile organic compound detection at room temperature and at a microwave frequency of 2.4 GHz. The gas-sensor response was defined as a phase difference of the sensor’s transmission coefficient measured under exposure to target gas and air. The developed gas sensors exhibited cross sensitivity to VOCs and thus could be realized as a gas-sensor array for selective target-gas detection. The CuO-based gas sensor had a good selectivity and sensitivity to acetone and practically no sensitivity to ethanol and methanol. However, both dioxides TiO_2_ and SnO_2_ could be applied for methanol detection; the gas-sensor response under methanol exposure was almost the same. For ethanol detection, the SnO_2_-based gas sensor exhibited the highest responses. It has to be underlined that the deposited metal oxides were deposited with various thicknesses and without any doping. The gas-sensing characteristics could be improved by changing the thickness (looking for the optimal thickness), changing the morphology, and by adding dopants, for example, Au, Pt, and Pd. Such experiments will be conducted in the near future, since microwave-based gas sensors are a very attractive solution in gas-sensing applications as they can be applied in portable electronics such as smartphones and tablets. In particular, the presented measurement system features high power efficiency and simple circuitry, since no frequency mixing is needed and, therefore, no conversion loss is obtained, which makes the presented system even more attractive for application in battery-supplied devices.

## Figures and Tables

**Table 1:** Metal oxides for gas-sensing applications deposited by magnetron sputtering technology.

Metal Oxide	Magnetron Sputtering Mode	Target Gases	Operating Temperature (°C)	References
CdO	DC	NH_3_	150	[13]
Co_3_O_4_	RF	CO	200	[14]
CuO	DC	NO_2_	200	[15]
	MF	C_3_H_6_O	450	[16]
Ga_2_O_3_	RF	O_2_	1000	[17]
In_2_O_3_	RF	CO/NO_2_	25	[18]
Fe_2_O_3_	RF	C_3_H_6_O	375	[19]
MoO_3_	DC	H_2_S	280	[20]
NiO	RF	H_2_	200	[21]
		NH_3_	300	[22]
Nb_2_O_3_	RF	CO	350	[23]
TeO_2_	RF	NO_2_	90	[24]
			25	[25]
SnO_2_	RF	NO_2_	60	[26]
	DC	NO_2_	150	[27]
TiO_2_	RF	H_2_	500 25	[28] [29]
WO_3_	DC RF	C_3_H_6_O CO	450 200	[30] [31]
V_2_O_3_	DC	CH_4_	25	[32]
ZnO	RF	H_2_S H_2_	250 75	[33] [34]
ZrO_2_	RF	O_2_	500	[35]

Notes: DC—direct current; MF—medium frequency; RF—radio frequency.

## Supplementary Materials

The following are available online at https://www.mdpi.com/1996-1944/12/6/877/s1, Figure S1: The copper oxide deposition system based on the magnetron sputtering technology, Figure S2: The titanium dioxide deposition system based on the magnetron sputtering technology, Figure S3: Tin dioxide deposition system based on the magnetron sputtering technology.
