# Supplementary material for: Metal Oxide Thin Films Prepared by Magnetron Sputtering Technology for Volatile Organic Compound Detection in the Microwave Frequency Range
Source: Materials (Basel). 2019 Mar 15;12(6):877. doi: 10.3390/ma12060877 (PMC6471264; doi:10.3390/ma12060877)
Supplement: Supplementary file 1 [file materials-12-00877-s001.pdf]

Supplementary Materials

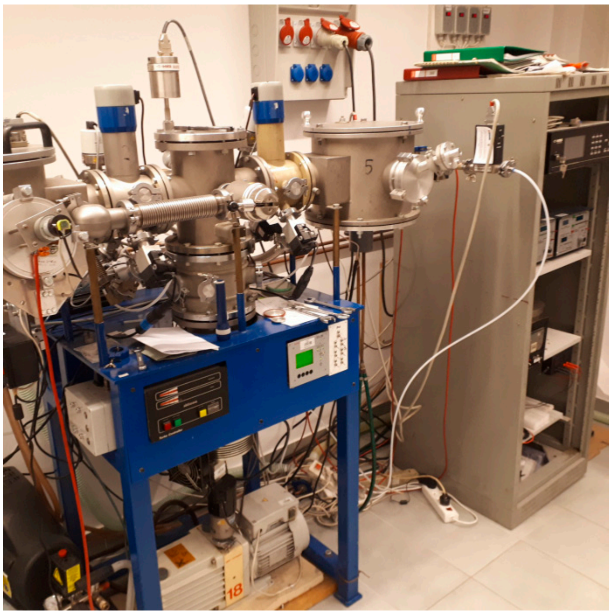

Figure S1. The copper oxide deposition system based on the magnetron sputtering technology.

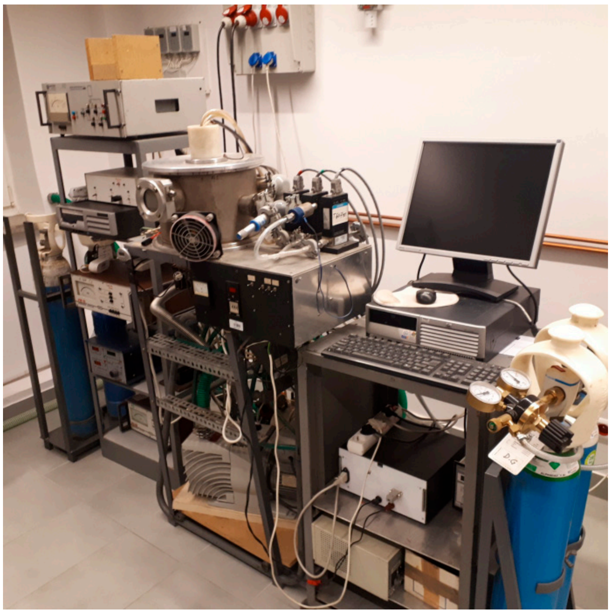

Figure S2. The titanium dioxide deposition system based on the magnetron sputtering technology.

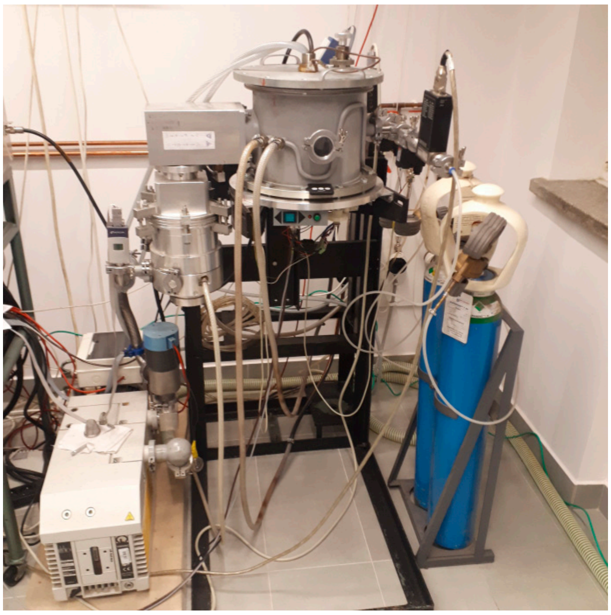

Figure S3. Tin dioxide deposition system based on the magnetron sputtering technology
